# Feasibility and potential benefits of defining the internal gross tumor volume of hepatocellular carcinoma using contrast-enhanced 4D CT images obtained by deformable registration

**DOI:** 10.1186/s13014-014-0221-7

**Published:** 2014-10-16

**Authors:** Hua Xu, Guanzhong Gong, Hong Wei, Lusheng Chen, Jinhu Chen, Jie Lu, Tonghai Liu, Jian Zhu, Yong Yin

**Affiliations:** Department of Radiation Oncology, Shandong Cancer Hospital and Institute, Laboratory of Radiation Oncology, Shandong Academy of Medical Sciences, 440 Jiyan Road, Jinan, 250117 China

**Keywords:** Four-dimensional computed tomography, Hepatocellular carcinoma, Radiotherapy, Deformable registration

## Abstract

**Objective:**

To study the feasibility and the potential benefits of defining the internal gross tumor volume (IGTV) of hepatocellular carcinoma (HCC) using contrast-enhanced 4D CT images obtained by combining arterial-phase (AP) contrast-enhanced (CE) 3D CT and non-contrast-enhanced (NCE) 4D CT images using deformable registration (DR).

**Methods:**

Ten HCC patients who had received radiotherapy beforehand were selected for this study. The following CT simulation images were acquired sequentially: NCE 4D CT in free breathing, NCE 3D CT and APCE 3D CT in end-expiration breath holding. All 4D CT images were sorted into ten phases according to breath cycle (CT_00_ ~ CT_90_). Gross tumor volumes (GTVs) were contoured on all CT images and the IGTV_-1_ was obtained by merging the GTVs in each phase of 4D CT images. The GTV on the APCE 3D CT image was deformably registered to each 4D CT phase image according to liver shape using RayStation^TM^ 3.99.0.7 version treatment planning system. The IGTV_-DR_ was obtained by merging the GTVs after DR on the 4D CT images. Volume differences among the GTVs and between the IGTV_-1_ and the IGTV_-DR_ were compared.

**Results:**

The edge of most lesions could be definitively identified using APCE 3D CT images compared to NCE 4D and 3D CT images. The GTV volume on APCE 3D CT images increased by an average of 34.79% (*P* < 0.05). There was no significant difference among the GTV volumes obtained using NCE 4D and 3D CT images (*P* > 0.05). The GTV volumes after DR on 4D CT different phase images increased by an average of 36.29% (*P* < 0.05), as was observed using the APCE 3D CT image (*P* > 0.05). Lastly, the volume of IGTV_-DR_ increased by an average of 19.91% compared to that of IGTV_-1_ (*P* < 0.05).

**Conclusion:**

NCE 4D CT imaging alone has the potential risk of missing a partial volume of the HCC. The combination of APCE 3D CT and NCE 4D CT images using the DR technique improved the accuracy of the definition of the IGTV in HCC.

## Background

The liver shape and position vary significantly during breathing. Indeed, it has been shown that liver motion can reach 0.5 ~ 5.0 cm during breathing [1-3]. Therefore, it is imperative to accurately determine the location of hepatocellular carcinoma (HCC) to improve the accuracy of radiotherapy [4]. Indeed changes in liver position could adversely affect radiotherapy planning and treatment, which could lead to inaccurate definition of the volumes of tumor and normal tissues, errors in dosimetric calculations and potential increases in radiation toxicity [5-7]. Therefore, eliminating the position uncertainty derived from breathing motion could improve the accuracy of HCC radiotherapy administration and therefore the clinical outcome of cancer patients [8].

Breath motion management methods for precision radiotherapy of HCC include active breathing control (ABC), abdominal compression and four-dimensional computed tomography (4D CT) techniques. In essence, dynamic 4D CT images could reflect the movement and deformation regularity of liver and tumors by sorting the volumetric CT images according to breath cycle which are recorded and segmented applying a respiratory position management (RPM) system [9,10]. Application of 4D CT in HCC radiotherapy has shown promising clinical outcomes. However, a contrast-enhanced (CE) 4D CT scan is lacking because of the difficulty of capturing the timing of contrast agent injection during the extended 4D CT image acquisition process [11]. In addition, breath motions bring more artifacts to all phases of non-contrast-enhanced (NCE) 4D CT image reconstruction [12]. It is well known that CE 3D CT image acquisition during the arterial phase yields a clear tumor edge for HCC [13]. Therefore, improving the detection methods to determine the location of the HCC target would improve the accuracy of radiotherapy for HCC.

Medical image deformable registration (DR) technique might prove a feasible approach in the treatment of HCC patients. DR could achieve a point-to-point correspondence between two given images, namely the objective image and the reference image, by voxel deformation and translation. The desired correspondence would describe the location of each tissue element in the first image relative to the second image [14]. DR has become the core technique of adaptive radiotherapy and recent studies have shown that it can both improve the accuracy of delineation of tumors and organs at risk (OARs) based on multi-modality imaging and assess the accumulation dose [15-18].

In this study we investigated the feasibility and benefits of defining the individual internal gross target volume (IGTV) of HCC using CE 4D CT images obtained by combining APCE 3D CT and NCE 4D CT images using deformable registration (DR).

## Materials and methods

### Patient information

Ten HCC patients (5 men, 5 women; ages 58 ~ 72) were studied at the Shandong Cancer Hospital following the guidelines of the ethics committee of the institution. The study were approved by the Research Ethics Board of the Shandong Cancer Hospital. All patients provided informed consent at enrollment and had their disease confirmed by radiography. None of the patients received transcatheter arterial chemoembolization (TACE). Of the ten tumors studied one was located in the left lobe of liver and the rest in the right hepatic lobe. Patients’ Karnofsky performance status was higher than 80 prior to receiving initial radiotherapy.

### CT acquisition

CT acquisition was performed using a Brilliance Big Bore CT scanner (Philips Medical Systems, Highland Heights, OH) with patients in a supine position. Scans (NCE 4D CT scans in free breathing, NCE 3D and APCE 3D CT scans in end-expiration breath holding) were acquired sequentially. 4D CT images were sorted into 10 series of CT images (CT_00_ ~ CT_90_) according to the respiratory phase, with CT_00_ being defined as the end inspiration and CT_50_ the end expiration. All CT images were exported into the RayStation treatment planning system (TPS), version 3.99.0.7 (RaySearch Laboratories, Sweden) for determination of GTVs, IGTVs, liver volumes and DR.

### Target acquisition

GTVs and liver contours were delineated on all images. IGTV_-1_ was acquired by merging the 10 GTVs at all phases of 4D CT images. The GTV on APCE 3D CT image was deformably registered to each phase of 4D CT images and labeled as _-DR_. The IGTV_-DR_ was obtained by merging the 10 GTVs_-DR_ after registration. The volume differences amongst the GTVs and between IGTV_-1_ and IGTV_-DR_ were compared. All GTVs and liver contours were delineated by the same radiation oncologist and reviewed by a second radiation oncologist for accuracy. To reduce inconsistency in the contours, standardized window settings were used for the liver (W/L 350/ 50).

### DR analysis and evaluation

DR analysis was performed using MORFEUS, a finite element model (FEM)-based multi-organ deformable image registration method that has been developed by RayStation TPS [19]. In this study, the APCE 3D CT image in end-expiration breath holding was selected as the target image and 10 phases of 4D CT images were selected as the reference images respectively. The target image was registered to different 4D CT phase images. The overall registration was performed in the CT region which included the size of the entire patient’s external body. The local registration was processed by tracking the liver volume as the organ of interest. After registration, the GTV on APCE 3D CT image was mapped to all phases of 4D CT images and 10 GTVs_-DR_ were generated. Brock *et al.* evaluated the accuracy of the algorithm and reported that the precision of the vessel bifurcation identification was less than 0.1 cm in the left-right (LR), anterior-posterior (AP) and superior-inferior (SI) directions, with the accuracy of the DR of the liver being lower than 0.2 cm in each direction [19].

### Statistical analysis

The data were analyzed using the SPSS 17.0 software package (IBM, Chicago, IL). The GTVs obtained from the different scans before and after DR were compared using one way analysis of variance. The paired *t-*test was used for the group comparison of GTVs and IGTVs before and after DR. A *P-*value <0.05 was considered statistically significant.

## Results

### Comparison of GTVs before DR

**Figure 1:**
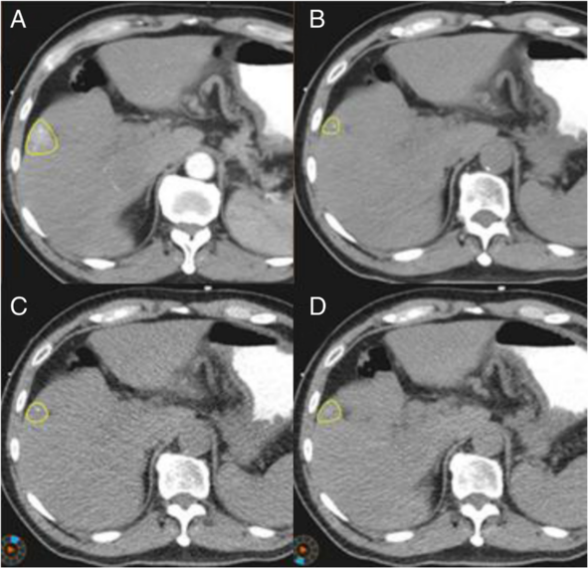
**GTV contours obtained from 3D or 4D CT scans before DR (on axial view). A**. contrast-enhanced 3D CT image. **B**. non-contrast-enhanced 3D CT image. **C**. CT_00_ respiratory phase of 4D CT images. **D**. CT_50_ respiratory phase of 4D CT images. Yellow contours: GTVs before DR.

As shown in Figure 1, the GTV on APCE 3D CT scan identified a clear edge and the boundary of the GTV could be easily determined. In contrast, the boundary was blurred on NCE 4D CT and 3D CT images. The average GTV from APCE and NCE 3D CT scans was 20.92 ± 13.20 cm^3^ and 15.52 ± 11.86 cm^3^ respectively and 15.13 ± 12.04 cm^3^ for NCE 4D CT scans. The GTV volume was 34.79% higher in APCE 3D CT image than in NCE 3D CT image (*P* < 0.05). No significant differences were found among the GTVs obtained using NCE 3D and 4D CT scans (*P* > 0.05), as shown in Table 1.

**Table 1:** **A comparison of GTVs obtained from 3D and 4D CT scans in different breathing phases before and after DR (** $\overline{x}$ **± S)**

**Scan/phase**		**GTV before DR (cm** ^**3**^ **)**	**GTV after DR (cm** ^**3**^ **)**	***t***	***P***
3D CT_CE_		20.92 ± 13.20	_	_	_
3D CT_NCE_		15.52 ± 11.86	_	_	_
4D CT_NCE_	CT_00_	15.03 ± 12.74	20.82 ± 13.41	-3.61	0.006
	CT_10_	14.84 ± 12.66	20.81 ± 13.65	-3.74	0.005
	CT_20_	14.72 ± 13.11	20.72 ± 13.66	-3.74	0.005
	CT_30_	14.94 ± 13.14	20.68 ± 13.70	-3.79	0.004
	CT_40_	14.93 ± 12.70	20.62 ± 13.24	-3.65	0.005
	CT_50_	15.66 ± 12.13	20.48 ± 13.13	-4.22	0.002
	CT_60_	15.17 ± 11.76	20.41 ± 13.25	-3.62	0.006
	CT_70_	15.03 ± 11.87	20.45 ± 12.71	-4.19	0.002
	CT_80_	15.43 ± 12.78	20.57 ± 13.36	-3.48	0.007
	CT_90_	15.50 ± 13.23	20.65 ± 13.36	-3.17	0.011
*F*		8.17	15.319	_	_
*P*		0.002	0.000	_	_

DR: deformable registration; CE: contrast enhancement; NCE: non-contrast enhancement; CT_x_: respiration phase.

### Comparison of GTVs after DR

**Figure 2:**
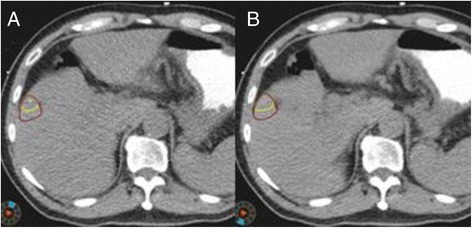
**DR improves the determination of GTV contours obtained from 4D CT images (on axial view). A**. CT_00_ respiratory phase of 4D CT image. **B**. CT_50_ respiratory phase of 4D CT images. Yellow contours: GTVs before DR; red contours: GTVs after DR.

After DR, the volumes of GTVs obtained from 4D CT images increased by an average of 20.62 ± 12.73 cm^3^, a 36.29% increase from the value obtained before DR (*P* < 0.05). The mean GTV after DR did not vary significantly from the value obtained from the APCE 3D CT image (*P* > 0.05), as shown in Table 1 and Figure 2.

### Comparison of IGTVs before and after DR

**Figure 3:**
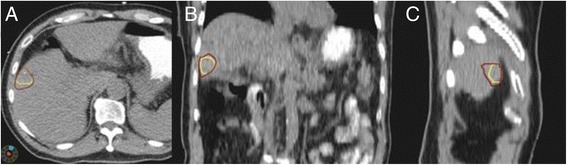
**A comparison of IGTVs before and after DR in 4D CT images. A**. axial view. **B**. coronal view. **C**. sagittal view. Yellow contours: IGTV before DR; red contours: IGTV after DR.

The mean volume of IGTV_-1_ before DR was 23.71 ± 16.67 cm^3^, while the mean IGTV_-DR_ after DR was 28.43 ± 16.09 cm^3^ with an average increase of 19.91% (*t* = -2.92, *P* = 0.017), as shown in Figure 3.

### DR analysis

**Figure 4:**
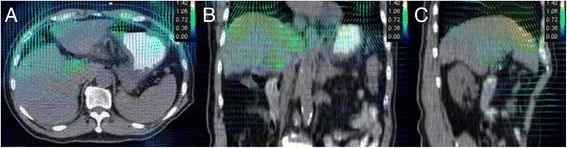
**Deformation fields for tumor and liver from contrast-enhanced 3D CT images to different respiratory phases of 4D CT images. A**. axial view. **B**. coronal view. **C**. sagittal view in CT_00_ respiratory phase. The variation of arrows and color show the vector fields on the axial, coronal and sagittal slices.

The image registration transformation vectors that were used to register the APCE 3D CT image to CT_00_ phase of 4D CT images are shown in Figure 4. The vector fields on the axial, coronal and sagittal slices are marked according to the legend. Ideally, the deformed CT_00_ phase image would be identical to the 3D CT image. In practice, it was similar but not identical due to imaging artifacts and spatial resolution inaccuracies. Inspection of the deformed regions suggests good agreement of the tumor and liver after DR, as shown in Figure 2.

## Discussion

The tumor target can move and deform significantly during respiration, making the accurate definition of liver lesions a critical step for high-precision radiotherapy [20,21]. 4D CT imaging is increasingly being used in radiation oncology to account for the effect of breathing on organ and tumor position in the thorax and abdomen. With this technique, the patient can breathe naturally during the 4D image acquisition process, making the therapy more comfortable. Therefore, 4D CT scanning in HCC radiotherapy could improve the accuracy of IGTV definition, reducing the margins of planning target volumes, and sparing more normal liver tissue from radiation. In summary, the 4D CT scanning technique would improve the accuracy of dose delivery and potentially result in better clinical outcomes for patients [22,23].

Conventionally, arterial-phase enhancement and venous-phase “washout” images are used in liver cancer patients to diagnose HCC [24]. In this study, we found that the tumor areas on the NCE 3D and 4D CT scans were not accurately seen due to a lack of image contrast, which makes the determination of tumor boundaries difficult and impairs the precise delivery of radiotherapy. In addition, we found that the volumes of GTVs obtained using enhanced 3D CT scans were on average higher than those obtained using non-enhanced CT scans, consistent with previous reports [25]. Currently, CE 4D CT scanning is not fully developed [26]. When the patients are administered complete TACE, the location and extent of the lesions on 4D CT images can be determined with the help of iodized oil. In contrast, when the patients receive incomplete TACE or initial treatment alone, there is a greater uncertainty in the definition of tumor volumes based on NCE 4D CT images alone [27]. Therefore, if the GTV generated on an artifact-free APCE 3D CT image could be mapped to all phase images of 4D CT with DR, some of the drawbacks of target delineation could be overcome.

MORFEUS allows for the image registration of both single and multiple organs. The latter can be challenging because of the difficulty of modeling the interface between organs using splines, fluid-flow and optical flow. This DR algorithm based on FEM allows the organs to be explicitly deformed by assigning the material properties and determining the boundary conditions. For some organs, including the liver, the boundary representation is consistent between different imaging modalities although the internal image intensity representation may vary. The feasibility and accuracy of MORFEUS was validated on MR thoracic and abdominal images from healthy volunteers at inspiration and expiration. For the lung and liver deformation, the average accuracy as measured by tracking visible bifurcations in the LR, AP and SI directions was 0.19 cm, 0.28 cm and 0.17 cm, respectively, with an average accuracy vector magnitude of 0.44 cm [19]. A FEM-based algorithm has been previously described for single organ DR. In a recent study, Ferrant *et al.* used this algorithm to register 3D intraoperative MR images of the brain and they reported that surface-based landmark discrepancies were reduced from 1.0 cm to less than 0.1 cm [28]. Likewise, Liang *et al.* tested the algorithm on a simulated deformation of the rectum and found that the error in registration was reduced from 0.9 cm based on an initial boundary point correspondence to 0.16 cm after optimization [29]. Zhang *et al.* also used this algorithm in a deformable lung model based on negative surface pressure [30]. Together, these reports support the accuracy and reliability of organ registration using MORFEUS even with internal organ motion and deformation.

Based on the above, the actual geometric verification of the mapped GTVs on 4D CT images, such as the shape and position of the GTV volumes, had not been performed. Apparently, the conventional way to verify the geometric change of the registered volumes is to introduce additional images, such as MR images, as a reference. MR images can yield high soft-tissue resolution. However, they require a long acquisition time and are susceptible to respiration and gastrointestinal peristalsis making their acquisition challenging. Moreover, a certain fusion error could inevitably exist when registering the MR and CT images. 4D MR would be expected to enable direct measurement of the tumor motion without contrast.

In this study, DR was performed in different series of images with the same imaging modality. Specifically, a CE breath-holding CT image was obtained followed by a 4D CT image. Therefore, the CT image sets had the same coordinate system and patient position. Thus, the registration error would be acceptable for the target delineation and mapping analysis. The GTV on APCE 3D CT images was deformed to match all the respiratory phases of the 4D CT images. The IGTV obtained after DR contained more tumor motion information and further improved the delineation accuracy of the tumor. To ensure the registration accuracy of whole liver, all 3D CT scans were acquired with the patient holding breath so the liver contours correlated with those on the single-phase 4D CT images. Compared with the IGTV generated from NCE 4D CT images, the IGTV obtained after DR can significantly increase the accuracy of target definition.

Beddar *et al.* [26] investigated enhanced 4D CT imaging of liver tumors using synchronized intravenous contrast agent injection. Before scanning, the patient’s intravenous line was connected to an automatic injector. The timing of the enhancement phase was programmed during 4D CT image acquisition to obtain good visualization of the tumor. However, the method was invasive due to the intervention with iodine oil. In addition, the optimal time window was difficult to capture as the automatic program would not work well with all patients. In contrast, as the DR technique is incorporated into modern TPS, the technique used in our study could be easily applied.

In summary, here we combined conventional target delineation protocols with image post-processing techniques to improve the accuracy of the definition of the IGTV of HCC using 4D CT. We showed that this protocol was more accurate in the determination of tumor boundaries. In future studies, we will evaluate the physiological impact of 4D radiotherapy administration.

## Conclusion

Here we show that contrast-enhanced 4D CT images, acquired by combining arterial-phase contrast-enhanced 3D CT and non-enhanced 4D CT images based on the DR technique could improve the definition of the IGTV of HCC and have the potential ability to improve the success of radiotherapy.
